# Inferring Mathematical Equations Using Crowdsourcing

**DOI:** 10.1371/journal.pone.0145557

**Published:** 2015-12-29

**Authors:** Szymon Wasik, Filip Fratczak, Jakub Krzyskow, Jaroslaw Wulnikowski

**Affiliations:** 1 Institute of Computing Science, Poznan University of Technology, Poznan, Poland; 2 European Center for Bioinformatics and Genomics, Poznan University of Technology, Poznan, Poland; University of Waterloo, CANADA

## Abstract

Crowdsourcing, understood as outsourcing work to a large network of people in the form of an open call, has been utilized successfully many times, including a very interesting concept involving the implementation of computer games with the objective of solving a scientific problem by employing users to play a game—so-called crowdsourced serious games. Our main objective was to verify whether such an approach could be successfully applied to the discovery of mathematical equations that explain experimental data gathered during the observation of a given dynamic system. Moreover, we wanted to compare it with an approach based on artificial intelligence that uses symbolic regression to find such formulae automatically. To achieve this, we designed and implemented an Internet game in which players attempt to design a spaceship representing an equation that models the observed system. The game was designed while considering that it should be easy to use for people without strong mathematical backgrounds. Moreover, we tried to make use of the collective intelligence observed in crowdsourced systems by enabling many players to collaborate on a single solution. The idea was tested on several hundred players playing almost 10,000 games and conducting a user opinion survey. The results prove that the proposed solution has very high potential. The function generated during weeklong tests was almost as precise as the analytical solution of the model of the system and, up to a certain complexity level of the formulae, it explained data better than the solution generated automatically by Eureqa, the leading software application for the implementation of symbolic regression. Moreover, we observed benefits of using crowdsourcing; the chain of consecutive solutions that led to the best solution was obtained by the continuous collaboration of several players.

## Introduction

Among the many interesting trends associated with the development of technology, we can distinguish two that result directly from its popularization. First, the rapid increase in the number of computers, mobile devices, sensors, and other electronics has caused an exponential increase in the amount of data collected, which, unfortunately, is not accompanied by an equally rapid development in techniques for data processing and knowledge discovery [1, 2]. Second, increasingly more people are wasting increasingly more time on the Internet performing activities that are not essential to life, coincidentally contributing to the data generation process. For example, according to 2012 research [3] approximately 32% of Facebook members use it to waste time, and 10% use it to access applications integrated within the portal. Considering that 89% of people with Internet access at work waste at least 30 min daily on tasks unrelated to their jobs, spending 23% of this time on Facebook [4], and including the fact that there are three billion Internet users, this adds up to millions of hours wasted on the Internet. If someone could utilize even a minute fraction of this human time to analyse data gathered on the Internet by exploiting the technique used by many crowdsourcing services, it could bring many benefits, especially considering that, as observed by Francis Galton in 1907, the collective opinion of a crowd of individuals can be much more precise than the opinion of a single expert [5].

The trends described above are probably the main reason for the rapid growth in the popularity of crowdsourcing. The term “crowdsourcing” was introduced in 2006 by Jeff Howe [6]. However, the concept of crowdsourcing, understood as outsourcing work to a large network of people in the form of an open call, is quite old. One of its first applications was when the British government offered prizes for the discovery of a method for measuring a ship’s longitude in 1714 [7]. Since that time, the concept of crowdsourcing has been utilized many times, but the rapid progression of such techniques started with the development of the Internet in the 1990s. The best examples of its results are services such as Wikipedia or OpenStreetMap. An extended review of crowdsourcing systems on the WWW can be found in [8] and a discussion about the nature of crowdsourcing can be found in [9].

An interesting application of the crowdsourcing concept is in the implementation of computer games whose objective is to solve some scientific problem by playing the game. These games, which are called crowdsourced serious games [10], already have several uses in the solution of problems from various fields of science. One of the first successful attempts to implement a crowdsourced serious game was Foldit, which asks players to fold a protein to obtain its tertiary structure. Players are awarded points according to the number of biological and chemical constraints satisfied. The results obtained by the players were so good that they have even been collectively included as co-authors of the paper published in *Nature* as “Foldit Players” [11]. Another biological application, EteRNA, asks players to design RNA sequences that fold into a target shape. This game proves that both the community of players and the algorithm created by analysing their behaviour could outperform all other known methods [12]. Another useful example of a crowdsourcing game is Eyewire, whose objective is to map a network of connections between neurons based on microscopy data. The project has already achieved initial success by detecting areas that respond to localized motion [13]. Finally, there are games developed by Verigames.com that aim to use crowdsourcing to perform formal verification of the software. Among the games available from Verigames.com, the most interesting from the perspective of our research is Xylem, which attempts to use players with low mathematical skills to define loop invariants that are in fact mathematical formulae. Unlike most such games, Xylem is available not only for the PC but also as a mobile game for the iPad [14].

The great success of crowdsourcing in so many disciplines suggests that it could also be successfully applied to the automatic discovery of models to explain data. For many centuries, scientists and mathematicians have attempted to find natural laws that explain the world surrounding them and models of various dynamic systems. Given the development of artificial intelligence, especially with regard to evolutionary algorithms [15, 16], there is little wonder that these algorithms also found application in an automatic search for mathematical equations that model various systems. In particular, some algorithms have recently appeared that successfully utilize symbolic regression analysis [17] to find such equations. One of the most successful is the Eureqa software application [18], which has been successfully used to solve many industrial and scientific problems. Other implementations include GPTIPS, a genetic programming and symbolic data mining platform for MATLAB [19], and RGP, a genetic programming framework for R that supports symbolic regression [20]. There have also been some successful attempts to model dynamic systems represented by differential equations [21–23]. However, all the above approaches are limited to rather simple systems because of the high computational complexity of the methods implemented [24]. Moreover, these approaches often tend to discover overfitted models because the fitness function usually prioritizes models that are more accurate rather than those with lower complexity [25].

The main objective of the present study was to verify whether crowdsourcing could be successfully applied to the discovery of mathematical equations that explain data gathered from dynamic systems. To achieve this goal, we designed and implemented a game in which players attempt to design a spaceship that represents an equation that models a given system. Additional objectives during the design of the game were to prepare a game targeted at people without advanced mathematical backgrounds and to make use of the collective intelligence observed in these crowdsourced systems. The game was tested by several hundred players in almost 10,000 games. Finally, we compared the results with the approach based on symbolic regression and analysed the users’ opinions of the game.

## Materials and Methods

### Application design

**Fig 1:**
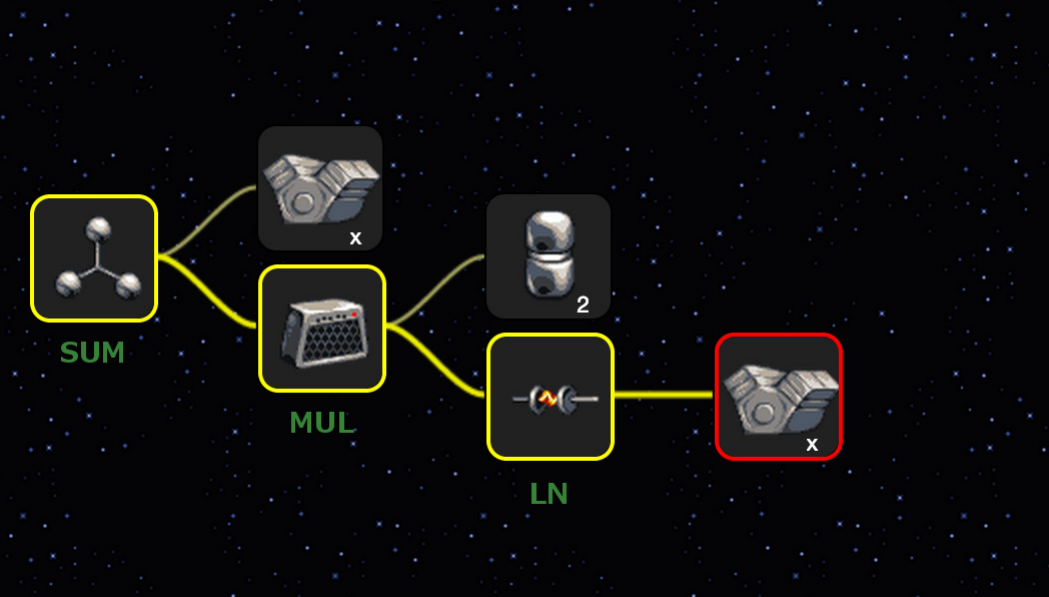
Example solution constructed by the player. The tree of upgrades to the spaceship represents the function *f*(*t*) = *t* + 2 ln *t*, which is the mathematical model of the system. In the figure *x* represents time (*t* in the function equation), and the value of the function models how the system behaves in time; however, the player does not have to know this. Green labels were added after the screenshot was taken to better explain how the solution was generated.

The game was designed as a web application, “Throw the hamster” (available at http://hamster.ovh/; example gameplay is presented in the S1 Video), which allows players to take part in the modelling of a dynamic system based on data integrated inside the game. The modelling is conducted by playing a simple game in which the player must design a spaceship for the hamster. Users can build new solutions from scratch or by modifying other users’ work. The flight trajectory of the spaceship depends on the design proposed by the user, and its objective is to fly as close as possible to stars placed in the sky. The spaceship flies from left to right, with its x-coordinate representing time and stars representing data points collected from some experiment. A model of the system is represented by the design of the spaceship in the form of a tree whose nodes consist of upgrades added to the spaceship. The tree structure is directly mapped into the expression tree [26] of the mathematical equation that models the system. Players can add, modify and remove the nodes. Each element affects the final trajectory. An example tree is presented in Fig 1. Players can test the designed structure at any time by shooting the hamster in the spaceship. After the hamster’s flight ends, the player is awarded points based on the accuracy of the equation. It is also possible to share solutions on social networks, which potentially encourages new players to visit the website.

If the tested solution turns out to be one of the best, it appears in the ranking list. Ranked models are publicly available for everyone to see. Moreover, anyone can use them for further modifications and, thus, incrementally construct a new solution based on them. As a result, players do not need to build their own models from scratch; they can improve the most accurate solutions, thus enabling them to boost the score creatively but with minimal work and time invested.

### Objective functions

Each solution prepared by players was evaluated via an objective function. For this purpose, we used a standard mean absolute error defined using the following formula:

$$\begin{array}{c} score=\frac{1}{N}\sum_{i=1}^{N}\left|y_{i}-f\left(x_{i}\right)\right| \end{array}$$ (1)

Here, *N* denotes the number of points (stars), *y*_*i*_ denotes the value of the data point recorded at time *x*_*i*_, and *f*(*x*_*i*_) is the value predicted by the player’s solution. As the solution becomes more accurate, the value of the objective function decreases, eventually reaching a very small value close to 0. The scoring procedure defined in Eq 1 is not very intuitive for users without mathematical backgrounds, so we proposed a transformation that guarantees three assumptions that are important from the perspective of usability. To make it easily understandable to players, the score should (1) increase as the solution improves, (2) not have negative values, and (3) be an integer. To guarantee these criteria, we defined a function that will always return a positive number less than or equal to 10,000:

$$\begin{array}{c} result=\left\lfloor \frac{10000}{max(\sqrt{score},1)}\right\rfloor \end{array}$$ (2)

Another function that characterizes the solution was used to measure its complexity and compare it with the results of the Eureqa software (described later). That is why we used the method of calculating the complexity of the mathematical formulae used in Eureqa. We assigned a weight to each of the operations that could appear in the formula and then summed the weights of all operations. The weights used by us are the same as the default weights used in the Eureqa software, and they are presented in Table 1. The value of the function that measured complexity was not presented to the players.

**Table 1:** Weights of particular operations used to measure the complexity of the solution.

Operation	Weight	Operation	Weight
addition	1	negation	1
subtraction	1	exponentiation	5
multiplication	1	logarithm	4
sine	3	natural logarithm	4
cosine	3	division	2
tangent	4	constant	1
cotangent	4	variable	1

### Testing

The testing procedure was conducted based on the data on Hepatitis C Virus (HCV) infections provided by Dahari et al. [27, 28]. These data describe how the viral RNA level decreases during therapy with pegylated interferon alpha and ribavirin. Because the analytical solution to the system of differential equations provided by Dahari is quite complex, we decided to attempt an automatic method to find the equation that explains the data.

#### Schedule and quantities

The tests were conducted using three iterations. After each iteration, the feedback from users was collected, the game design was analysed, bugs were fixed, and some new features were implemented. The statistics summarizing each iteration are presented in Table 2.

**Table 2:** Number of games, players and browser sessions in successive test iterations. Games played equals number of hamster launches. The time column presents the total time spent by all players on playing the game. The average duration of a single game is equal to 29.8 s.

Iteration	Players	Sessions	Games played	Games per user	Time
#0	∼20	∼40	∼400	∼20	∼3.3 *h*
#1	616	928	7,628	12.38	63.1 h
#2	90	212	1,525	16.94	12.6 h
**Total**	**726**	**1,180**	**9,553**	**13.16**	**79 h**

The preliminary iteration of tests (#0) consisted of internal tests on a small group of players. It helped to detect several bugs and a few problems related to the user experience. Statistics for this iteration are only estimations because bugs in the code and frequent database updates prevented their precise calculation. The first large-scale iteration (#1) was the first major test. The main objective of this iteration was to verify the proposed concept. The game was published on the Internet and presented to a wider group of people. We found several minor bugs, but, most importantly, we identified several misconceptions in the game’s design. We corrected them and proceeded to the final iteration of tests (#2). These final results were also compared to the output of the Eureqa software. Details on the results are presented in the following sections.

The study involved Internet users who played the online game and were recruited by messages published on social networks. All statistics used during the research were collected anonymously, and all players were informed such that, according to Polish law, there was no need to collect consent from participants or obtain approval from the institutional review board. The authors did not have access to any potentially identifying information at any point of the study (including user IP addresses).

#### Reference results from Eureqa

We used version 0.99.9 of the Eureqa software, which was the most recent version when the experiment was conducted. The search was executed using the default values of parameters and the same mathematical operations, complexity weights and objective function as in the final iteration of the game (#2). The search was executed for 18 h using four cores of a 64-bit i7 CPU and, during that time, evaluated 1.5 × 10^11^ formulae.

### Users opinion survey

During the two large test iterations, a survey was carried out to collect feedback on the game. The survey consisted of seven questions. We asked players how they liked the game and what they wanted to change about it. We also checked whether they were aware that this game was designed not only to be fun for the players but also to solve an important scientific problem. Finally, we asked about the players’ attitudes toward mathematics to check whether there was a correlation between mathematical skills and the results in the game. Fifty-seven players out of 726 completed the survey. Detailed questions and all collected answers are presented in S1 Table, and results are discussed in the following sections.

### Implementation

The main objective of the technical part of the project was to provide the application on the largest possible number of platforms. The game was implemented using the latest portable technologies, including HTML 5, CSS 3, Javascript, PHP and MySQL. Signing in to the game supports integration with Facebook and Google+ accounts and anonymous access. All screens are created according to a single-page-application pattern to provide a fluid user experience. The backend of the game stores all solutions in the database, which provides advanced analytical functions using the SQL language. Every solution has the structure of a tree, in which nodes are mathematical operations, variables or constant values. The tree is stored in JSON format, thus supporting interoperability.

## Results

As presented in Table 2, during the first phase of tests, we attracted many more players. However, during the second phase, the players were much more dedicated to the game; on average, each of the players constructed 37% more solutions, so the results had higher quality instead of quantity. Some of the players in iteration #2 were repeats from the first phase; however, most of them were new players. Usually, players who constructed a small number of solutions did not find good formulae because the process of improving them is complex and requires time. The following sections contain a detailed analysis of each iteration of tests. Detailed results from all games that were played are presented in S2 Table.

### Generated functions

**Fig 2:**
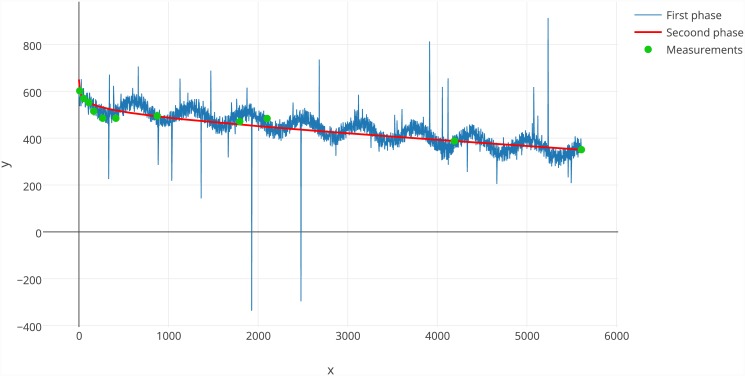
Comparison of the best functions obtained in each phase. In the first phase, the influence of trigonometric functions is easily visible. Moreover, when the denominator of the component containing the cosine function approaches 0, we can observe spikes in function values.

Fig 2 presents the best functions from both the large-scale testing phases and the values of experimental data points. During the first test phase, players experimented significantly with trigonometric functions. Using trigonometric functions with a small period and large amplitude, it was possible to cover a wide range of points. The solution actually consisted of multiple overlapping sines and cosines. The objective function for such a solution has a high value, but a related modelling problem usually expects a simpler form of the equation. To solve this problem, we forbade the use of trigonometric functions during the second iteration of tests. After removing these functions, the best solution found by players was much simpler, and it does not overuse trigonometric functions.

### Comparison with Eureqa

The following contains a comparison of the best functions constructed by players in each test iteration with results found by the Eureqa software. Eureqa does not find a single solution but a set of them; it stores the best solution for each level of formula complexity. From all of these solutions, we compared the best one with the best among all with a complexity less than or equal to the complexity of the solution constructed by players during the second phase of tests. For each function, we present the mean absolute error, the score presented to players in the game, and the complexity of the function. The functions can usually be simplified using basic arithmetic operations, but we avoided this to present the raw form of the formula generated by players or Eureqa.

#### Iteration #1: first testing phase

The best solution constructed by players. Mean absolute error: **4.93**. Game score: **4,510**. Weighted complexity: **65**.

$$\begin{array}{c} \begin{array}{cc} f(x)= & 20\cdot \sin\left(0.01\cdot 20\cdot \log\left(1+x^{29.9969}\right)\cdot x\cdot \left(-0.1\right)\right)+\left(-0.036\cdot x\right)+\\ & \frac{20\cdot \cos(0.01\cdot x)}{0.585}+20\cdot \sin\left(0.01*60.3\right)+20\cdot \cos\left(0.001\cdot x^{2.98}\right)+\\ & 532.44+\left(\frac{-24.8}{20\cdot \cos(0.01\cdot x^{1.555})}\right)+0.0051\cdot \left(-1\cdot 20\frac{\log\left(1+x\right)}{log\left(2\right)}\right) \end{array} \end{array}$$ (3)

#### Iteration #2: second testing phase

The best solution constructed by players. Mean absolute error: **14.63**. Game score: **2,614**. Weighted complexity: **23**.

$$\begin{array}{c} \begin{array}{cc} f(x)= & 649.23+\left(-1.019\cdot 20\frac{\log\left(1+x\right)}{\log(2.715)}\right)+\\ & (-0.022\cdot x)+1.13+x\cdot x\cdot 0.0001\cdot 0.0001\cdot 0.2 \end{array} \end{array}$$ (4)

#### Eureqa #1

The best solution found by Eureqa with complexity less than or equal to the best solution from the second iteration of tests. Mean absolute error: **20.37**. Game score: **2,215**. Weighted complexity: **23**.

$$\begin{array}{c} f\left(x\right)=100\left(4.4+\frac{3.5}{\frac{x}{100}+2}-0.01\frac{x}{100}+0.01\cdot 4.4\right) \end{array}$$ (5)

#### Eureqa #2

The best solution found by Eureqa. Mean absolute error: **5.93**. Game score: **4,104**. Weighted complexity: **72**.

$$\begin{array}{c} f\left(x\right)=100\left(2+4.4-\sqrt{\sqrt{0.01+{\frac{x}{100}}^{2-\frac{x}{100}\cdot 2\cdot 0.3^{2}}+\frac{{\frac{x}{100}}^{2}\cdot 0.3^{2}}{2^{2}}}}\right) \end{array}$$ (6)

The best result was obtained during the first phase of tests thanks to the use of trigonometric functions, which outperformed even the best solution generated by Eureqa. In a much more realistic case modelled during the second iteration of tests, the result is much worse; however, it is still slightly better than the result generated by Eureqa for the same complexity. Nevertheless, when not constrained by the complexity level, the result generated by Eureqa is better than the solution constructed by the players. The reason for this is probably the problem pertaining to the handling of complex solutions by players, which is later discussed in detail.

The time spent on calculating solutions in both approaches was comparable. Eureqa calculated the solution in approximately 18 h. In the second half of this time, improvements to the solution were very small, and at the end of this time, the solution stopped improving. Iteration #1, measured as the total time spent by all players on playing the game, was approximately three times longer and equal to approximately 63 h. However, in iteration #2, when more dedicated players were playing the game, the total time spent on solving the problem was a few hours shorter and equal to approximately 13 h. Details are presented in Table 2.

### Gameplay analysis

#### Best solution formation

**Fig 3:**
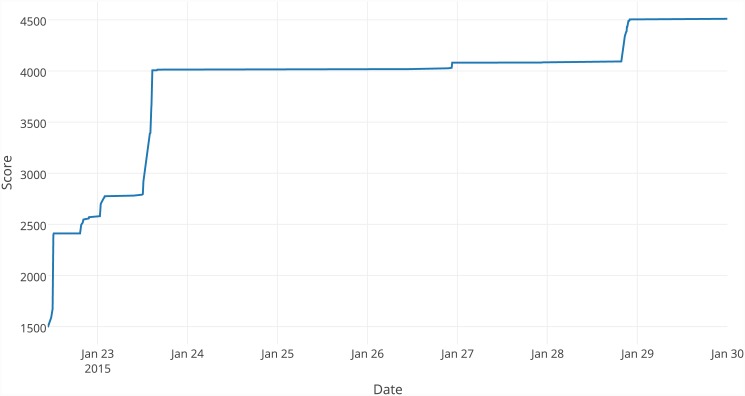
Formation of the best solution during the first testing phase.

**Fig 4:**
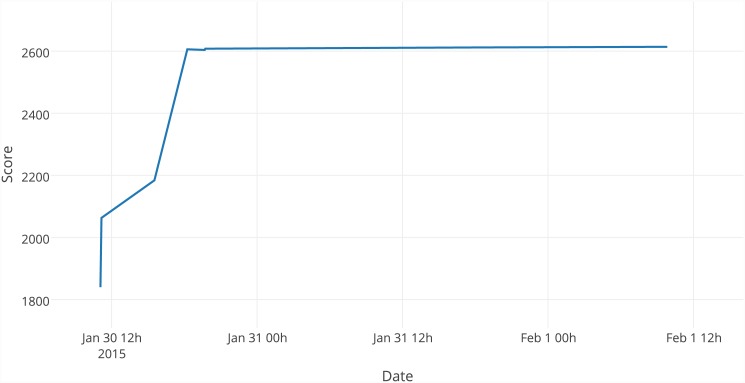
Formation of the best solution during the second testing phase.

Figs 3 and 4 present how the value of the best solution changed over time as it was improved by players during the first and second phases of the tests. During each phase, there was a sharp increase in the value of the objective function over the first day of tests. During the first day of the first iteration, the solution was improved at four points, which are significantly connected with the addition of consecutive trigonometric functions. During the second phase of tests, it was more difficult to improve the solution without trigonometric functions; as a result, there are fewer improvements.

#### Highest increments

**Fig 5:**
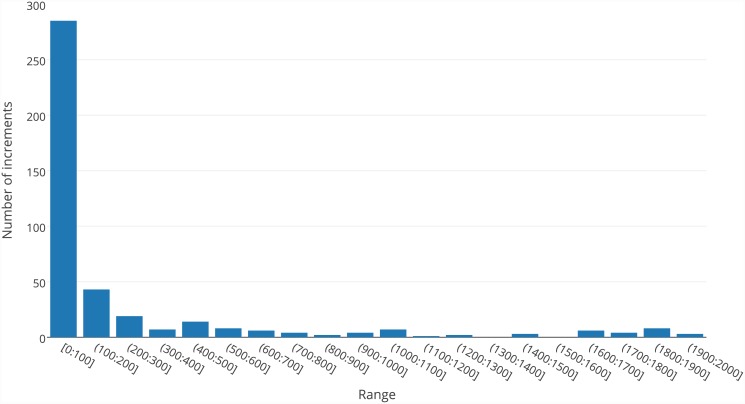
Number of score increments in each range.

Most of the players constructed their solutions based on some other solution—their own solution or one of the best solutions constructed by other players. We define an increment as the difference between the score of the new solution and the base score. Fig 5 presents the number of increments in each score range during the first phase of tests. The results for the second phase are similar. We present the results for the first phase because, during the second phase, fewer games were played.

Most increments were not high and were in the range of 0–100 points. Only a few players modified their functions in such a way as to achieve a result that was much higher than the base solution (with a score of at least 300 points higher). This is consistent with the analysis of the process on how the best solution was constructed (detailed data can be found in S3 Table). The best solution was created using 104 small improvements, usually by increments of fewer than 20 points, created by 17 different players. This is perfect proof of the collaborative nature of the process that led to the construction of the final solution.

#### Solution complexity

**Fig 6:**
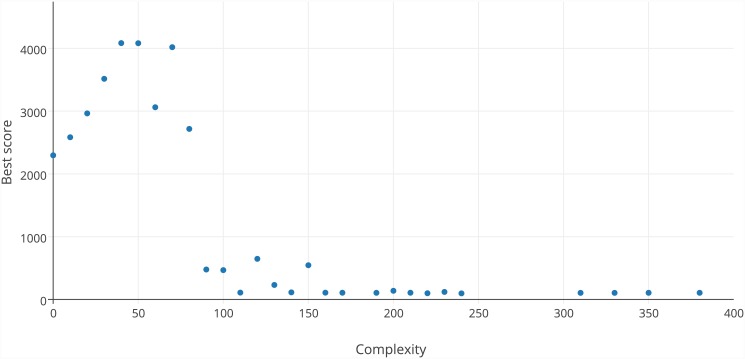
Best score for each solution complexity.

Fig 6 shows the best score for solutions constructed at each level of complexity during phase #1. The analysis of increment results for phase #2 was similar. For complexity in the range between 0 and 40, the value of the objective function is proportional to the complexity. This is quite obvious because increasing complexity allows the construction of more complex formulae that can be better fitted to data. For complexities between 40 and 70, the score does not change, and for complexities greater than 70, it is very difficult for users to design an effective solution. This is why, for larger complexities, Eureqa software outperforms the presented approach.

### User opinion analysis

The survey confirmed that the appropriate game design and method for sharing information about the game ensured that people were aware of the scientific objective of the game. Eighty-two percent of them confirmed that they read the description explaining the scientific background of the game, and another 16% admitted that, although they had not read it, they were aware that there was some scientific aim. Many players declared that they played the game several times regardless of the fact that the game was not very interesting to them. This could suggest that they really understood the significance of the game and continued to play because of the scientific objective. Moreover, most players were more likely to recommend the game to others than not.

The survey also allowed the formation of some ideas about improvements that could be introduced to the game. Many people complained that they did not understand how adding upgrades to the spaceship influences its flight. This problem was partially solved during the second phase of tests by adding a description to each of the upgrades, thus explaining its influence and significantly improving the reception of the game during the second phase. In this iteration, for each upgrade, we provided the mathematical operation that it represents and an explanation for people with lower mathematical knowledge’for example, *“Sum—aggregates behaviour of connected elements”*, *“Subtraction—amplifies difference in behaviour of two elements”*. According to the survey, we also failed to attract users with lower mathematical skills; 95% of players declared an interest in mathematics.

All collected opinions are presented in S1 Table.

## Discussion

The objective of the research was to verify whether it is possible to use a crowdsourced game to solve the problem of finding mathematical formulae to explain experimental data. To answer this question, we implemented a simple web game and integrated it with social networks. Based on the large number of games (almost 10,000), we can conclude that the verification was successful. The group of people could, in a relatively short time, construct a solution better than that found by the leading software application that uses artificial intelligence algorithms based on symbolic regression. However, it should be noted that both solutions work only for the discovery of a formula of a single equation. Their application would be much more interesting if they could find formulae of a set of equations or, even better, a set of differential equations. Thus, it can be more easily used to explain how the system described by these equation works in reality. Nevertheless, these are much more complex problems that require separate consideration.

However, the artificial intelligence methods performed better for very complex formulae. The reason for this is probably the problem with controlling complex solutions manually by humans. This can be clearly seen in Fig 6. There is a complexity limit of 40, at which humans encounter difficulty in constructing good solutions, and another limit equal to 70, at which it is almost impossible to successfully process such a complex formula. Most of the users did not even try to construct such a complex solution, which is probably another reason why the value of the score for a complexity higher than 70 decreases dramatically. This difficulty can probably be solved by decomposition of the problem to smaller sub-problems—e.g., to design each component of the target formula separately. However, this creates new difficulties because such sub-problems are not independent, and the solution of one sub-problem influences how another sub-problem should be solved. These new difficulties in turn require a change in the values of data points based on the solutions of various sub-problems and, as a result, could require significant modification of the game’s design assumptions.

The key observed advantage of crowdsourcing is the collaboration of many players. The creation of the best solution was possible thanks to the cooperation of 17 players who constructed a chain of more than 100 improvements (see S3 Table). Some of them introduced just one improvement, and some of them introduced several improvements in a row; however, the most interesting is the contribution of users 09, 11 and 14. They played the game several times, each of which improved the solution based on other players’ contributions. The whole set of their actions presents behaviour similar to onsite collaboration in which several people are working together locally. However, thanks to online crowdsourcing, they did not have to be simultaneously collocated or even communicate with one another. The efficiency of the collaboration is also well presented in Fig 5, which proves that good solutions are usually constructed by a large number of small improvements created by different users.

Another interesting conclusion from the tests is the observation of how quickly players realized that using trigonometric functions could easily improve the solution’s score. This was an obvious error in the game design that was successfully corrected before the second phase of tests. It is also worth noting that, for players, the biggest problem in the game was understanding how a change in the design of the spaceship influenced its flight trajectory. According to the user survey, it was a problem or a large problem for 40% of players. This was partially solved before the second phase of tests by adding a more detailed description of each upgrade; however, this solution can be improved further to obtain better results by including some type of tutorial.

One of the important conclusions from the tests and user opinion survey is that the game itself should be more interesting and engaging to attract players. The many volunteers that participated in the tests of the game have already proved that the implemented approach could be successful from a scientific point of view. However, they emphasized that the game should be more attractive to players to stimulate them to play the game for a longer time. This is why we have currently suspended the search for new players and are preparing a new version of the game that will be more interesting for users to play while addressing the most serious concern observed during the tests—difficulties in understanding how the design of the spaceship influences its flight.

To summarize the article, we presented a novel approach for finding mathematical formulae that explain experimental data gathered from the analysis of dynamic systems. The solution is a crowdsourced serious game, which proved to be very successful in solving this problem. There are still some drawbacks that must be solved before widespread implementation of this method, but they were identified during the research and well defined, and we have some ideas on how to solve them. Currently, the game can be classified as a very difficult puzzle game, but adding some action elements to the simulation of the hamster’s flight could make it much more entertaining. The best proof of how a minimal, score-based action game can engage millions of players can be provided, for example, by the success of the simple Flappy Bird game [29]. After solving the identified drawbacks, it would also be interesting to test the game based on more datasets from various areas of science.

## Supporting Information

S1 Video Example gameplay. Video that demonstrates an example interaction with the game: construction of the spaceship, its flight and selecting another user’s solution as a starting solution to improve the spaceship.
(MP4)

S1 Table Survey results. The table contains all answers to the closed-ended questions inside the survey. It also contains the description of each question and possible answers. The information about the game was spread among our friends; that is why most of the open-ended questions were answered in Polish, so we do not include them. The analysis of answers to open-ended questions is included in the article.
(XLSX)

S2 Table Game results. Game results generated during the first and the second iterations of tests. Each row presents the solution designed by the player, its value, the player’s id and the base solution that was used.
(XLSX)

S3 Table Chain of improvements. Chain of improvements that lead to the best solution. It presents the sequence of solutions, each of which is based on the previous one with the value of the increment and id of the player.
(XLSX)
